# Study of Orally Disintegrating Tablets Using Erythritol as an Excipient Produced by Moisture-Activated Dry Granulation (MADG)

**DOI:** 10.3390/ph15081004

**Published:** 2022-08-15

**Authors:** Mizuki Yamada, Agata Ishikawa, Shun Muramatsu, Takayuki Furuishi, Yoshinori Onuki, Kaori Fukuzawa, Etsuo Yonemochi

**Affiliations:** 1 Department of Physical Chemistry, School of Pharmacy and Pharmaceutical Sciences, Hoshi University, 2-4-41 Ebara, Shinagawa-ku 142-8501, Tokyo, Japan; 2 Laboratory of Pharmaceutical Technology, School of Pharmacy and Pharmaceutical Sciences, University of Toyama, 2630 Sugitani, Toyama-shi 930-0194, Toyama, Japan; 3 Graduate School of Pharmaceutical Sciences, Osaka University, 1-6 Yamadaoka, Suita 565-0871, Osaka, Japan

**Keywords:** orally disintegrating tablet, erythritol, moisture-activated dry granulation, disintegration, water activity

## Abstract

Moisture-activated dry granulation (MADG) is an eco-friendly granulation method that uses a small amount of water and insoluble excipients to absorb moisture. MADG is expected to improve productivity and reduce costs. Erythritol, an excipient used for preparing orally disintegrating tablets (ODTs), has poor tabletability and is difficult to form into tablets by conventional methods, such as high-shear granulation (HSG) and direct compression. In this study, we optimized the manufacturing conditions for ODTs to improve the tabletability of erythritol using MADG. The disintegration time of tablets made using the MADG method was approximately one-tenth that of those made using the HSG method, and the hardness was approximately 1.4 times higher. Moreover, MADG could delay disintegration and improve tabletability. We further attempted to optimize the manufacturing conditions using MADG, particularly in terms of the amount of water used. The disintegration time increased as the amount of added water increased. Moreover, water absorption tests revealed that capillary wetting decreased as the amount of water added increased, but the initial wetting did not change. These results suggested that the disintegration time was prolonged because of the increase in granule density and decrease in capillary wetting with the increase in the amount of added water. The hardness of the tablets increased because of the easy deformation of the granules after the addition of up to 3% water; however, when more than 3% water was added, the hardness decreased because of the aggregation of the granules with the excess water. Finally, two-dimensional maps of the effect of the amount of added water and water activity indicated that tablets with a hardness of ≥80 N and a disintegration time of ≤15 s could be produced by adjusting the amount of added water to within the range of 2.2–3.3% and water activity to 0.3–0.53. These results indicate that MADG can improve the tabletability of erythritol and be used for the granulation of ODTs. Tablets with appropriate hardness and disintegration properties can be produced by adjusting the water content to approximately 2.7% and the water activity to approximately 0.4 when producing ODTs with MADG.

## 1. Introduction

Difficulty in swallowing, or dysphagia, is a growing health concern in an aging population. Age-related changes in swallowing physiology as well as age-related diseases are predisposing factors for dysphagia in the elderly. Although the exact prevalence of dysphagia across different settings is unclear, conservative estimates suggest that 15–22% of the elderly population is affected by dysphagia [1,2,3]. Patients are unlikely to seek advice from a health professional before splitting or crushing solid doses [4]. To improve patient medication management, we aimed to identify individuals who are more likely at risk of experiencing difficulties in swallowing medication.

Recent developments in dosing technology offer an alternative medication delivery method for patients with oropharyngeal swallowing problems [5,6]. Orally disintegrating tablets (ODTs) are a form of solid dosage that disintegrates rapidly upon contact with saliva in the oral cavity. Prescription ODT products were initially developed to overcome the difficulties experienced by pediatric, geriatric, and psychiatric patients with dysphagia in swallowing conventional tablets. ODTs offer an easy alternative form of intake not only for prescription drugs but also for over-the-counter (OTC) drugs, such as tablets and capsules, without water [7]. ODTs are preferred by patients over traditional dosage forms and are designed to disintegrate rapidly on the tongue. Disintegration should result in an easy-to-swallow, smooth suspension; ideally, ODTs should contain taste-masked drug microparticles to eliminate the bitter aftertaste. According to the US FDA Guidance for Industry Orally Disintegrating Tablets, ODTs should disintegrate within approximately 30 s of consumption, with no need for chewing or drinking liquids, i.e., patients should be able to consume them with or without water [8]. ODTs combine the advantages of solid dosage forms with novel formulation technologies to better fulfill patient needs; they are referred to as a patient-centric dosage form because of their ability to cater to specific patient groups. ODTs are ideal for administrating drugs to pediatric and geriatric patients. ODTs minimize dosage form errors and improve compliance and therapeutic outcomes because of their ease of use.

Wet granulation is one of the most widely used tablet manufacturing process to improving granule and tablet properties. In particular, high-shear granulation (HSG), wherein the granules are produced by adding water to the powder, mixing, and drying, is one of the most popular processes in the pharmaceutical industry as it leads to the production of easily flowing granules with the active pharmaceutical ingredient (API) being uniformly distributed. It also masks potentially unfavorable compression properties (e.g., aggregation, low flowability and so on) and also equilibrates the variabilities that may arise during drug development or because of sourcing APIs from different suppliers. From the processing aspect, HSG is inferior to direct compression or dry granulation because it involves an extra drying step after the agglomeration stage using separate equipment, such as a tray dryer or fluidized-bed dryer.

Water content is one of the most important parameters of wet granulation; many researchers have investigated the importance of the amount of added water quantities in HSG. Sherif et al. described the effect of different HSG parameters on granule characteristics, including granule size and compressibility, stating that the amount of added water had the greatest impact on granule size, density, and compressibility of granules [9,10]. Preeetanshu et al. also described the effect of different parameters of the HSG process on granule characteristics using experimental and modeling approaches and concluded that the amount of added water had the greatest impact on granule characteristics [11]. Limin et al. reported that the increased granule size was an important factor underlying over-granulation in HSG [12]. Therefore, the amount of water in the HSG process must be controlled. A disadvantage of HSG is that it has a higher production cost than direct compression and other dry granulation processes, as the drying process and intermediate products need to be transferred to other machines.

In the pharmaceutical field, direct compression (DC) has recently been widely used for manufacturing oral dosage tablets [13,14,15]. DC basically involves only three processes: milling, blending, and tableting. Only three main pieces of manufacturing equipment are required to produce tablets via DC: blending, sieving, and tableting machines. This process can reduce the effort involved in transferring intermediates to other machines and cleaning equipment. Therefore, DC is the simplest and most economical manufacturing method of producing oral dosage forms compared with wet and dry granulation methods [16,17]. DC is also beneficial for moisture and heat sensitive APIs because DC requires no granulation water or heating process [13,14,15,16,18,19]. The characteristics of an API have an impact on the final mixture and tablet properties of DC. Most APIs need a micronizing process to improve their solubility in water. However, micronized API has low flowability and high static electricity [20], leading to manufacturing problems and high variability in the content of DC. Functional excipients are available for improving the flowability and compaction properties of the commercial base; however, these excipients have little impact on high-drug-loaded DC formulations, especially for loading of over 20% or 30% [15]. In these cases, the granulation process is necessary to overcome undesirable API characteristics [21].

Moisture-activated dry granulation (MADG) is attracting attention as an innovative granulation method that combines the advantages of HSG with a new drying process. MADG was first reported by Ullah et al. in 1987 [22] as an eco-friendly manufacturing alternative to conventional methods of manufacturing oral dosage tablets. The whole process can ideally be performed within a conventional high-shear granulator, from pre-blending all components intended for granulation to the final blending of further functional excipients as disintegrants or lubricants prior to compression. Hence, the transfer of granule intermediates during processing to other equipment may not be necessary, thus saving time and electricity consumption. Finally, as transfer steps are the main source of unintended exposure, MADG is an ideal granulation process for the manufacture of solid dosage forms from highly potent compounds. In general, the MADG process can be divided into two process steps: the aggregation step and moisture absorption step [23,24]. After the granulation process, the moisture-absorption process is conducted in MADG instead of using heat. In this process, water-insoluble excipients such as microcrystalline cellulose (MCC) and colloidal silicon dioxide are usually used as moisture absorbents. The powder is agitated, whereby the water is reduced and uniformly distributed. In the first step, the main drug, excipients, and binders are placed in a stirring granulator and, in MADG, granulated by adding a very small amount of water (1–4%) instead of the conventional 15–20% [25]. In the next step, insoluble excipients such as MCC [26] and hygroscopic agents such as colloidal silicon dioxide are used to adsorb water; subsequently, water is reduced and uniformly distributed. Conventional methods involve drying with hot air; however, MADG does not require a drying process, thus reducing manufacturing time and energy requirements and eventually reducing costs. Another advantage of MADG is that it can be applied to heat-unstable pharmaceuticals. So far, previous studies have focused on MADG and related research [25,27,28,29,30,31,32,33,34].

Conventional manufacturing methods for ODTs include casting, wet tableting, and compression molding [35]; however, all these methods have high porosity and low density to facilitate rapid disintegration, resulting in low tablet hardness [36]. Therefore, it is necessary to improve the hardness while maintaining rapid disintegration [37]. Sugar alcohols, such as mannitol, are often used as excipients in the formulation of ODTs because of their good solubility and sweet taste when dissolved [36]. Erythritol is a sugar alcohol that is used as a food additive and sugar substitute and has almost zero calories. In the pharmaceutical field, erythritol is a promising excipient for ODTs because it provides a refreshing cold sensation and sweet taste when dissolved and has low hygroscopicity. However, erythritol has fewer polar hydroxy groups than other sugar alcohols, resulting in low surface free energy and poor compressibility [38]. Bi et al. used erythritol as an excipient in ODTs but found it difficult to produce tablets with high erythritol content because of its low tabletability [39]. Improving the tabletability of erythritol has therefore become a major issue in designing an ideal ODT.

In the present study, we attempted to improve the tabletability of ODTs, including erythritol, using MADG; compared MADG with the HSG and direct compression (DC) methods; and evaluated the important quality characteristics of the ODTs, including disintegration time and hardness. Using MADG, ODTs were prepared by varying the amount of water added to the formulation to optimize the manufacturing conditions.

## 2. Results and Discussion

### 2.1. Visual Observation of ODTs

**Figure 1:**
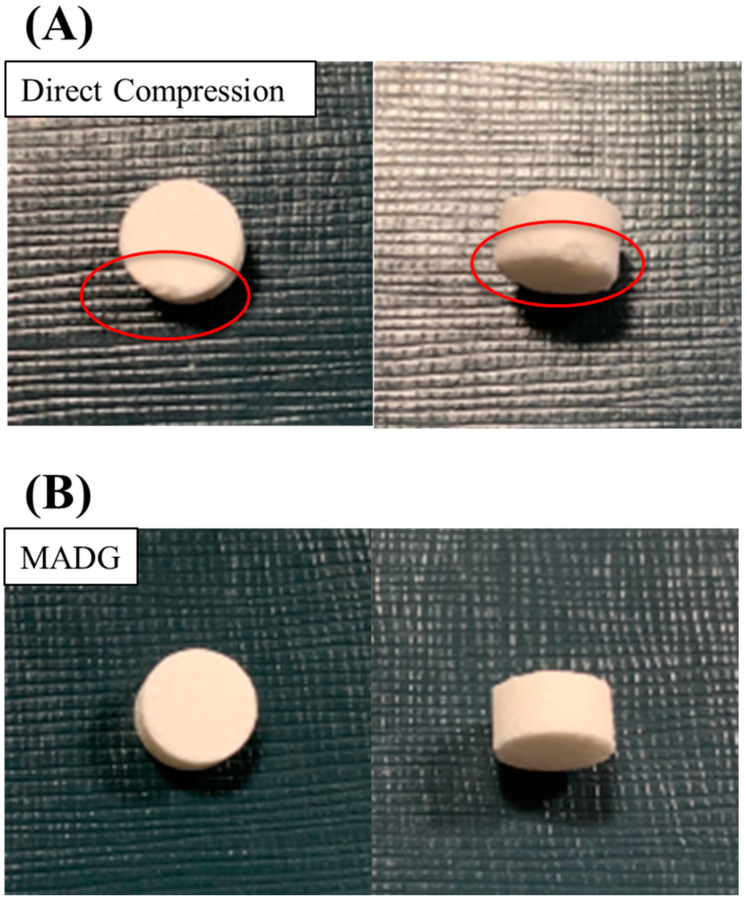
Photographs of ODTs produced using the (**A**) DC and (**B**) MADG methods.

As in a previous study [40], the amount of water added to ODTs prepared using MADG and HSG was set at 2% and 20%, respectively, but the tablets could not be formed using HSG because of sticking; therefore, tablets produced via HSG with 15% water were used for comparison. Tablets could be formed using MADG and HSG, but those prepared with DC had chipped corners (Figure 1A), indicating that the tabletability of erythritol is poor. We decided to compare MADG and HSG for further study.

### 2.2. Tablet Disintegration Time and Scanning Electron Microscope Photographs

**Figure 2:**
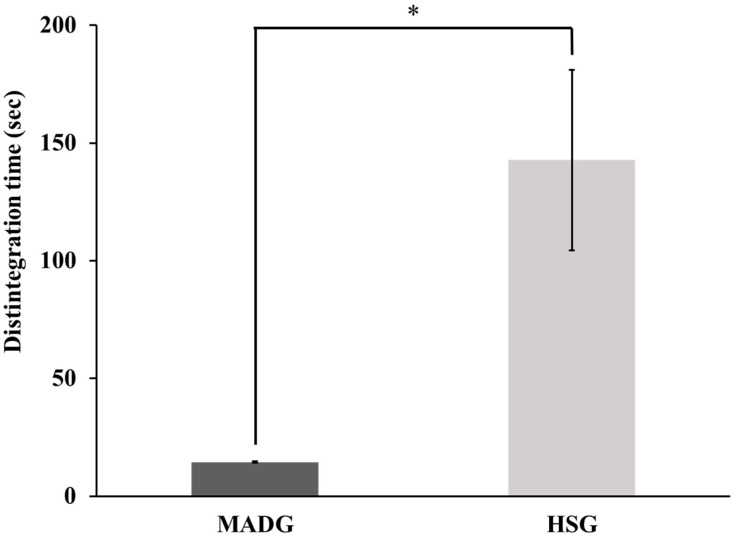
Disintegration time of tablets produced using MADG and HSG. Bar values are presented as average ± S.D. (*n* = 3). Student’s *t*-tests were used to determine the statistical significance of differences with respect to MADG. * *p* < 0.05.

Figure 2 shows the disintegration time of the tablets. The tablets prepared using MADG disintegrated in 14.5 s, and the tablets prepared using HSG disintegrated in 142.7 s; MADG disintegrated in approximately one-tenth of the time taken by HSG tablets. The MADG tablets met the criterion of a disintegration time of 30 s for ODTs. On the hands, friability was much less than 1% for MADG (0.72%) and HSG tablet (0.55%). The disintegration of tablets is related to the wettability and porosity of the granules [41]. MADG reportedly has a faster wicking speed and higher porosity of tablets than HSG [25]. The disintegration time of MADG tablets was approximately one-tenth of that of HSG tablets. Figure S1 shows the SEM photographs of granules by MADG and HSG. From the view of the photographs, the degree of surface deformation of the granules by HSG was higher than that of the granules by MADG so that the particle adhesion would be stronger. Figure S2 also shows the SEM photographs of inner structure of tablets by MADG and HSG. This result indicates that the non-uniform density of inner tablet by MADG was observed compared that of tablet by HSG. Moreover, a previous study shows that the mean particle size of MADG was smaller than that for HSG and tablet porosity produced by MADG showed much increase compared to the porosity produced by HSG [25]. Therefore, it was assumed that granules made of MADG conduct water more easily because of the high porosity and non-uniform density of the tablets, resulting in shorter disintegration time than HSG.

### 2.3. Tablet Hardness

**Figure 3:**
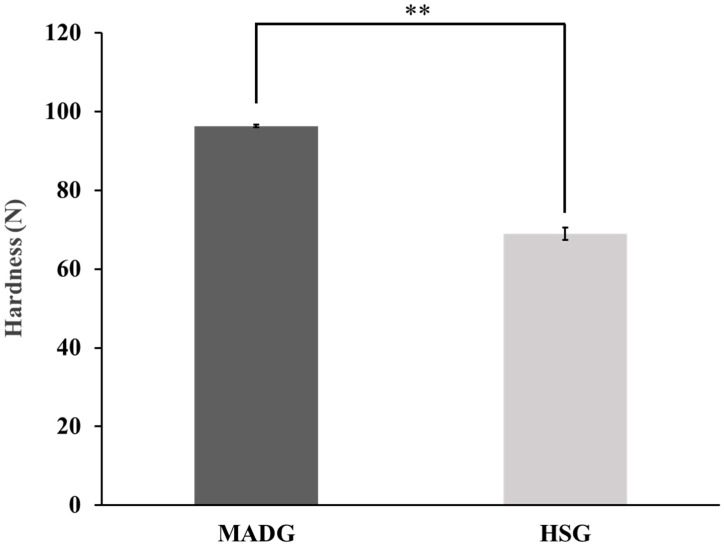
Hardness of tablets produced using MADG and HSG. Bar values are presented as average ± S.D. (*n* = 3). Student’s *t*-tests were used to determine the statistical significance of differences with respect to MADG. ** *p* < 0.01.

The results of the hardness test are shown in Figure 3. MADG showed 96 N, which is approximately 1.4 times higher than HSG (69 N). In both MADG and HSG, the compression moldability of ODTs including erythritol improved because of the MCC 200LM and PH102, which have high compression moldability [25]. Badawy et al. reported that wet granulation decreases the porosity of MCC, and the tabletability of MCC decreases when it agglomerates into large particles [42]. The difference in tablet hardness between MADG and HSG would be because of the decrease in porosity of MCC caused by granulation. As MADG needs much less water for granulation than HSG, the aggregation of granules may have decreased. As there was no drying process, thermal effects on the granules would be decreased and the porosity increased compared with HSG. HSG produced dense and spherical granules, which increased the flowability; however, the granules were dense, hard, and aggregated during granulation, suggesting that the tablet hardness of HSG was lower than that of MADG because of the loss of porosity of MCC. Developing tablets, easy to take and convenient to use, is important to improve patient adherence to medication. Additionally, simplifying the tablet manufacturing process is economically and environmentally important since it reduces manufacturing costs and saves energy. Erythritol is a promising excipient for ODTs because it has high solubility and a refreshing cold sensation and sweet taste when dissolved; however, it has low tabletability. Using the excipients with improved tableting properties supplied by manufacturers of pharmaceutical additives is one way to solve tableting problems but are expensive. Conventional manufacturing methods for ODTs such as casting, wet tableting, and compression molding require complicated manufacturing processes and produce tablets with low hardness. Therefore, application of MADG to ODTs is considered superior in terms of convenience, cost, and manufacturability because MADG can produce highly erythritol-loaded tablets with higher hardness than those of conventional manufacturing methods for ODTs and reduce the manufacturing process and energy consumption compared to conventional methods.

### 2.4. Effect of Water Content on the Water Activity of Granules Produced Using MADG

Water activity is defined as the percentage of free water in the granules. Because water activity has a large effect on granules in relation to the total water content [43], the water activity value of granules produced by MADG method was measured in this study. At 1% water addition, the water activity was 0.31. As the water content increased, the water activity increased, and at 4% water addition, the water activity was 0.57 (Figure S3). As water activity increases, porosity is lost and granules aggregate [44], which affects the Hausner ratio and granule size [33].

The water activity of the additives with high hygroscopicity was the lowest, with 0.34 for PVP and 0.38 for crospovidone. Erythritol and MCC, which are low hygroscopic additives, showed high water activities at 0.44 and 0.45, respectively (Figure S4). This suggests that PVP and crospovidone originally retain less free water. Therefore, they are capable of adsorbing a large amount of water, resulting in higher hygroscopicity. The water activity values of the granules could be regulated by adding lower hygroscopic additives (erythritol and MCC) to increase the water activity and by adding higher hygroscopic additives (PVP and crospovidone) to decrease the water activity.

### 2.5. Effect of Water Content on the Tabletability of ODTs Produced Using MADG

We prepared ODTs using MADG with different amounts of water. Tablets could not be formed when 1% and 5% water were added to the granules; this was because capping of the tablet occurred at 1% and sticking of the tablet at 5% (Figure S5). We therefore prepared ODTs using the MADG method with a range of 1.5–4.0% water.

### 2.6. Effect of Water Addition on Tablet Disintegration Time

**Figure 4:**
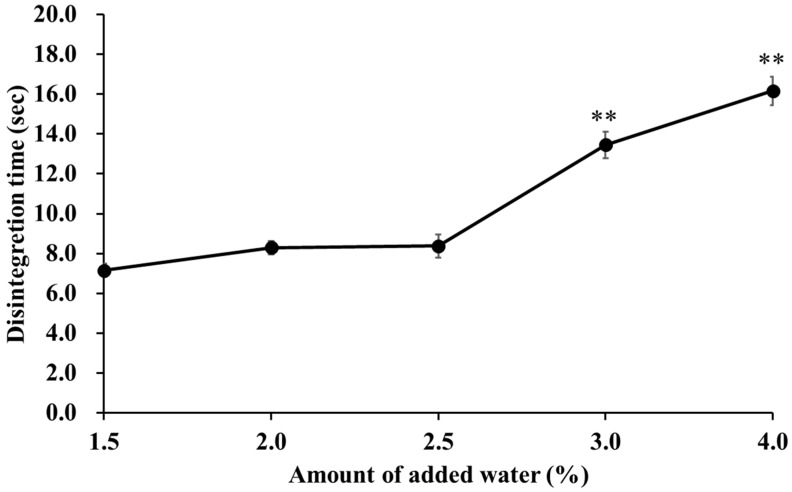
Effect of the amount of added water on the disintegration time. Each point indicates average ± S.D. (*n* = 3). Analysis of variance was used to determine the statistical significance of differences with respect to the 2.5% added water. ** *p* < 0.01.

Figure 4 shows the changes in tablet disintegration time with the amount of added water. The disintegration time was 7.15 s at 1.5% water content. However, the disintegration time increased with increasing water content, and the disintegration time at 4.0% water content was 16.16 s. We hypothesize that this phenomenon occurs because the density of the granules increased with increasing water content, which inhibited swelling and water penetration into the granules. We therefore carried out a water absorption test.

**Figure 5:**
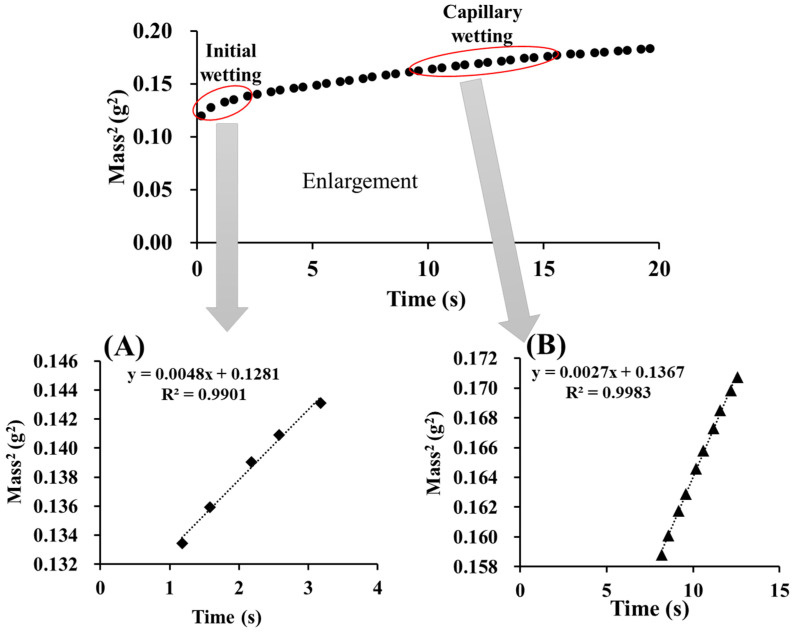
Water absorption profile: (**A**) initial wetting, (**B**) capillary wetting.

**Figure 6:**
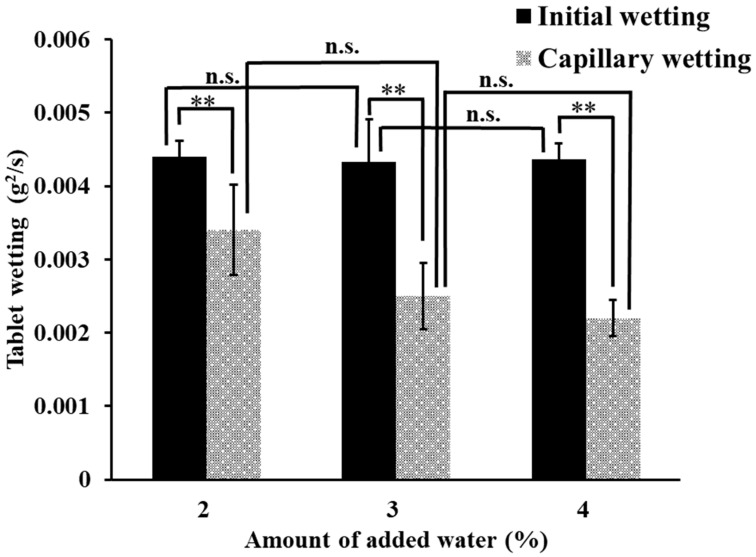
Volume of added water vs. tablet wetting. Each bar indicates the average ± S.D. (*n* = 3). Analysis of variance was used to determine the statistical significance of differences. ** *p* < 0.01; n.s., not significant.

The water absorption profiling results are presented in Figure 5. This profile suggests the presence of two wetting behaviors: initial wetting and capillary wetting. Initial wetting was defined as the wetting of the surface of the tablet bed. Subsequently, water penetrates the tablet in a process defined as capillary wetting. Initial wetting was calculated using a five-point linear regression method, whereas capillary wetting was calculated using linear regression at the equilibrium condition using 10 points [25]. Figure 6 also shows an enlarged image of the data obtained by a surface tensiometer, divided into initial and capillary wetting.

Figure 6 summarizes the initial wetting and the capillary wetting profiles obtained with different amounts of added water. The initial wetting did not change with increasing water content to approximately 0.0044 g^2^/s, whereas the capillary wetting was 0.0034 g^2^/s at 2% and decreased to 0.0022 g^2^/s at 4%. This result suggests that capillary wetting, not initial wetting, was responsible for the disintegration time. It was further clarified that the increase in water content resulted in increased density of the granules and decreased capillary wetting, thus confirming the prolongation of the disintegration time.

### 2.7. Tableting Properties

**Figure 7:**
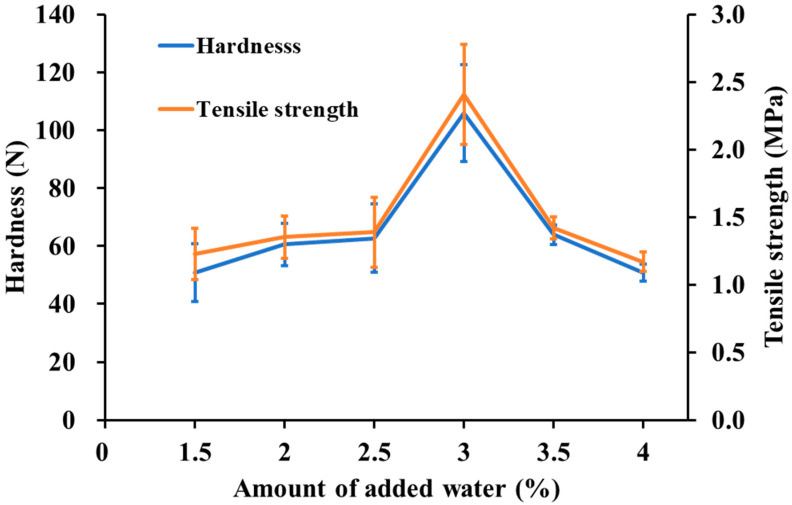
Effect of the added water on tablet hardness and tensile strength. The range indicated by the blue arrow is the range in which the hardness is >80 N and the disintegration time is within 15 s. Each bar (blue bar: hardness, orange bar: tensile strength) indicates the average ± S.D. (*n* = 3).

The results of the tablet hardness and tensile strength tests are shown in Figure 7. The tablet hardness of ODT produced using the MADG method was 50.77 N at 1.5% moisture content and increased with increasing moisture content, reaching a maximum value of 105.77 N at 3% moisture content. Although the capping of tablets at the addition of 1% water occurred, the increase in tablet hardness from 1.5% to 3% water was assumed to occur because the granules were easily deformed with the increase in the amount of water. However, the presence of excess water causes granules to aggregate and reduces the porosity [44], suggesting that the addition of >3% water decreases the flowability and tablet hardness because of granule aggregation. Moderate moisture content prevents the granules from agglomerating and provides better alignment during tableting, which may result in higher tablet hardness. The tensile strength was also calculated from the tablet hardness and showed the same trend as tablet hardness (Figure 7). Figure 6 shows the decreasing trend of capillary wetting with increasing addition of water; however, Figure S6 shows that there was no significant difference (*p* > 0.05) between 2% and 4% water addition, indicating that initial and capillary wetting did not affect the tablet hardness.

We next conducted two-way analysis of variance (ANOVA) on the tablet properties obtained from Gamlen Development Series: D500, which can measure pressure and displacement in the compression process, as well as the friction during ejection and the hardness of the resulting tablets [45,46,47,48,49]. The ANOVA tables and effect leverage plots for each characteristic are available as supplemental materials. From the ANOVA tables, it was confirmed that the loading weight had a significant effect on all the characteristics examined. Furthermore, these effects were far higher than those of the difference in the sample (Table S1a–e). As for effect of the difference in the sample, significant effects were observed from the characteristics except for compression pressure and tablet density. For further understanding of the mode of action of the factors on characteristics, the effect leverage plots were evaluated (Figure S7a–e). X and Y axes of the plots represent the value of characteristics and the leverage residuals for the characteristics, respectively. Regarding the plots for difference in the samples, the data on the left and right sides of the plots represent the data of HSG and MADG, while data distributing on the left, middle, and right sides on the plots of loading weight correspond with those of 200, 350, and 500 kg. As shown in Figure S7a–e, all plots showed positive slopes. This means that the characteristics increased with higher loading weight and the characteristics of HSG were lower than those of MADG. From these findings, we demonstrated that the tablets prepared by HSG and MADG were different from each other in terms of manufacturing and tablet properties.

### 2.8. Effect of the Amount of Added Water and Water Activity on the Disintegration Time and Tablet Hardness

**Figure 8:**
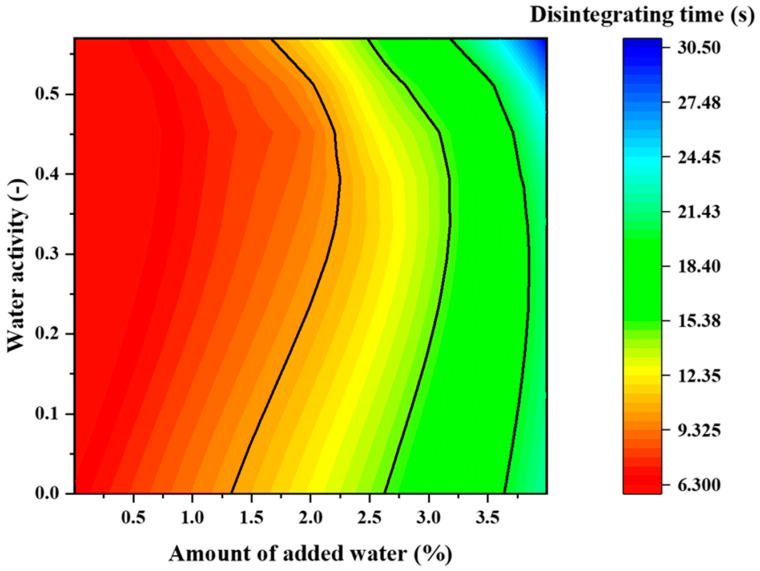
Two-dimensional map showing the effect of volume of added water and water activity on the disintegration time.

**Figure 9:**
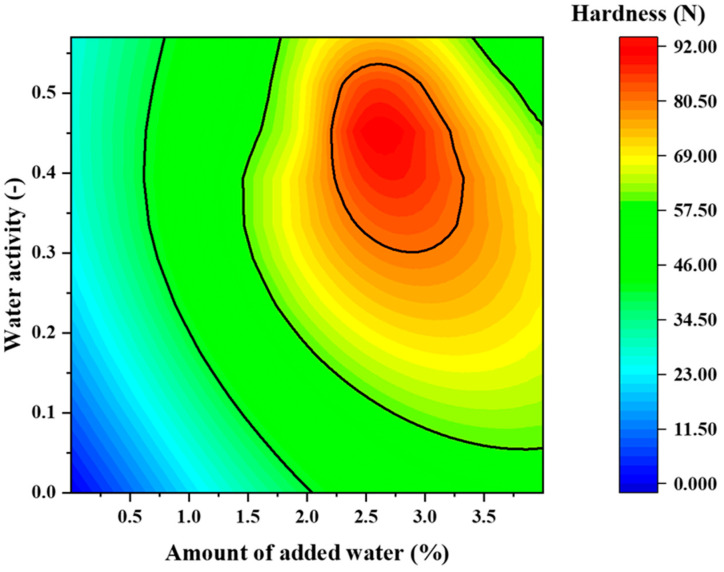
Two-dimensional map illustrating the effect of the volume of added water and water activity on tablet hardness.

**Figure 10:**
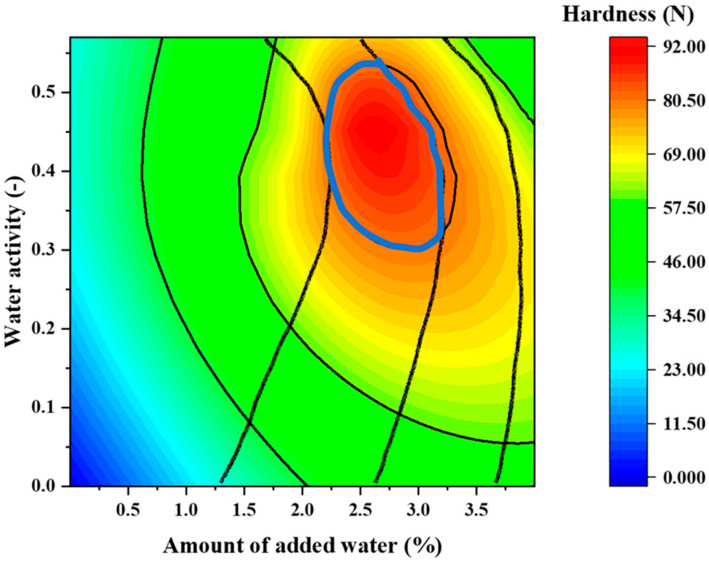
Two-dimensional map presenting the marked disintegration time of 15 s and hardness of 80 N.

Figure 8 presents a two-dimensional map of the relationship of disintegration time to water activity and water addition, and Figure 9 presents a two-dimensional map of hardness vs. water activity and water addition. In all ranges, the disintegration time was <30 s, which is the criterion for ODTs (Figure 8). The two-dimensional map of disintegration time is superimposed on the response surface of hardness in Figure 10. The range indicated by the blue arrow is the range in which the hardness is >80 N and the disintegration time is within 15 s. From this result, it is clear that tablets with high hardness and rapid disintegration can be produced by adjusting the water content to 2.2–3.3% and water activity to 0.3–0.53.

## 3. Materials and Methods

### 3.1. Materials

Erythritol (Lot: 16963) was purchased from Cargill Japan (Tokyo, Japan), PVP K12 (Lot: 86391588Q0) and K25 (Lot: 33580536W0) were purchased form BASF Japan LTD. (Tokyo, Japan), MCC, Avicel^®^ PH-200LM (Lot: LM1631C) and Avicel^®^ PH101 (Lot: LM1631C) were purchased from FMC (Philadelphia, PA, USA), Crospovidone XL (Lot: 0002022443) was purchased from Ashland Co Ltd. (Tokyo, Japan), and magnesium stearate (Lot: 16963) was purchased from Magnesia GmbH (Lüneburg, Germany). All solvents used were of analytical grade, and purified water was used in this study.

### 3.2. Preparation of ODTs Using MADG, HSG, and DC

**Figure 11:**
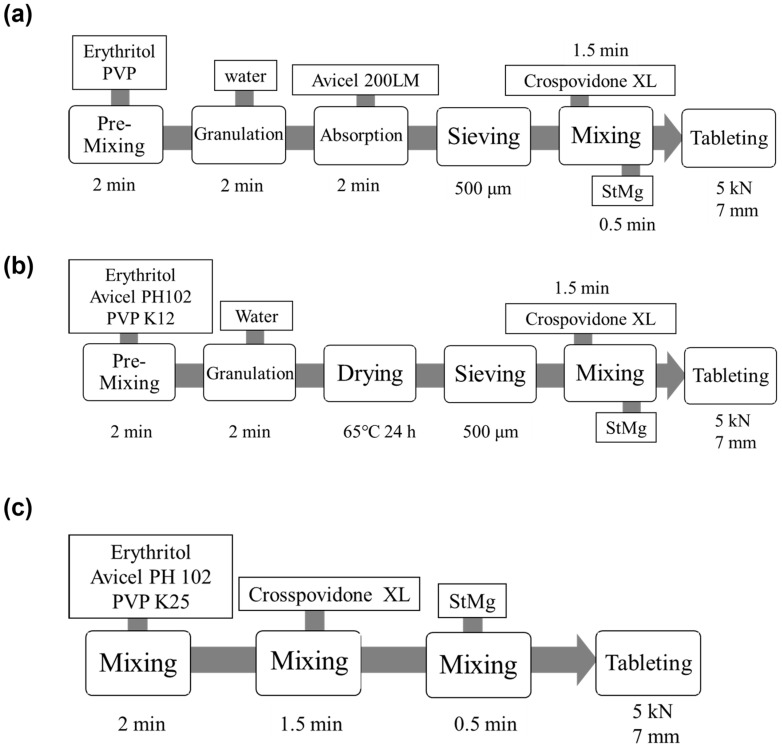
Manufacturing flow of ODTs using (**a**) MADG, (**b**) HSG, and (**c**) DC.

The formulation of granules and the process of the preparation of the samples using MADG method are presented in Table 1 and Figure 11, respectively. The following procedure was used: erythritol as an excipient and PVP as a binder were mixed for 2 min. Higher molecular weight PVP has been proven to be unsuitable for the MADG method [25]; therefore, PVP K12 was selected in this study. The amount of water added during granulation was varied from 1% to 5%, and after mixing for 2 min, MCC, a moisture absorbent substance, was added and mixed for 2 min. The grade of MCC was PH-200LM, as in a previous study [25]. After mixing and sieving through a 500 cm sieve, the granules were used as samples. In the granules made using the HSG method, erythritol and MCC PH102 as excipients and PVP K25 as a binder were mixed for 2 min with the addition of 15% water and then dried at 60 °C for 24 h. After the drying process, the granules were sieved through a 500 cm sieve. In the granules made using the DC method, MCC PH102, PVP K25 and erythritol were mixed for 2 min with the addition of 15% water and then dried at 60 °C for 24 h. Finally, crospovidone XL was blended in for 1.5 min, and then magnesium stearate was blended in for 0.5 min. A single punch press (P-165-028, RIKEN SEIKI Co., LTD., Nagano, Japan) was used to prepare flat-face tablets of 7 mm diameter and 200 mg of mass at a compression force of 5 kN.

### 3.3. Water Activity

The water activity of the final blends was measured using a water activity meter (EZ-200, FREUND CORPORATION, Tokyo, Japan) (*n* = 1).

### 3.4. Tablet Disintegration Time

The disintegration time of the tablets was measured at 37 °C using a disintegration tester (NT-4H, Toyama, Japan) at 30 cycles/min (*n* = 3). No discs were added, and distilled water was used as the test medium. The disintegration time was recorded as the time required for all particles to completely pass through the mesh screen of the apparatus.

### 3.5. Tablet Friability

This test was carried out according to the USP standards. A total of twenty randomly selected tablets (*n* = 33, minimal weight of 6.5 g of the tablets was weighed) were dedusted, weighed (W_1_), and placed in the friabilator (EF-2, Mumbai, India), which was then rotated 100 times for 4 min. After that, tablets were removed, dedusted, and weighed (W_2_). Tablet friability was determined as percentage loss of weight using Equation (1), where ≤1.0% loss in weight was considered acceptable.

Friability = (W_1_ − W_2_)/W_1_ × 100 (1)

### 3.6. Scanning Electron Microscopy (SEM)

Scanning electron microscopy (TM3030Plus Miniscope, Hitachi High-Tech Corporation, Tokyo, Japan) was used to evaluate the surface of the granules and tablets. Tablets were broken in half and the microscope was focused on the inside of the tablets. The SEM images were captured by electron beam accelerating voltage of 5 KV, 100 and 300× magnification, and 15 KV and 500× magnification, respectively.

### 3.7. Tablet Hardness

Tablet hardness was measured via diametrical compression tests using a tablet hardness tester (Minhua Pharmaceutical Machinery Co., Ltd., Shanghai, China) (*n* = 3). The tensile strength (σ_t_) was calculated using the following Equation (2) [50]:

σ_t_ = 2F/(πDT) (2)

where F is the tablet breaking force, D is the tablet diameter, and T is the tablet thickness.

### 3.8. Wettability of Moisture Absorbents and Tablets

The wettability of the tablets was measured using a surface tensiometer (K100; Krüss GmbH, Germany) at 25 °C. First, a tablet was placed in a stainless-steel tube, suspended into the probe, and lowered into the liquid (water). The time and weight of the liquid that penetrated the tablet were recorded. Wetting behavior includes initial and capillary wetting. Initial wetting was calculated using the first five data points in a linear regression, excluding the first point, whereas capillary wetting was calculated using linear regression. For the calculation of the initial and capillary wetting, the equilibrium part (R^2^ ≥ 0.95) of the coefficient of mass^2^ and time was used [25].

### 3.9. Tableting Properties

As manufacturing properties, compassion pressure, the detachment stress, ejection stress, tensile strength, and tablet density of the sample powders were measured using a benchtop single-punch dynamic powder compaction analyzer Gamlen Development Series: D500 (Gamlen Instruments, Nottingham, UK). This equipment monitors the forces of upper and lower punches during the compaction and ejection processes of the compressed tablet. Sample powders (100 mg) that had the same composition as the model tablet were filled into a die, 6 mm in diameter, and compressed into flat tablets by an upper punch at a speed of 60 mm/min. The load was set at 200, 350, and 500 kg. Compression pressure was calculated as follows in Equation (3):

Compression pressure (MPa) = P_c_/((r/2) ∗ (r/2) ∗ π) (3)

where P_c_ is the compression force and r is the punch diameter.

Afterwards, the lower punch was detached from the compressed tablet. The detachment force required to detach the lower punch from the tablet was measured, and the detachment stress was calculated as follows in Equation (4):

Detachment stress (MPa) = P_d_/(πr^2^) (4)

where P_d_ is the detachment force and r is the radius of the punch.

The resulting tablet was ejected from the die. The friction force between the die and the tablet, when it was ejected (ejection force), was monitored. Ejection stress was calculated as follows in Equation (5):

Ejection stress (MPa) = P_e_/(πrt) (5)

where P_e_ is the ejection force, r is the radius of the punch, and t is the thickness of the tablet, which was measured by a digital indicator (Model 543–782 Digimatic Indicator-ID-S1012MX, Mitutoyo Corporation, Kanagawa, Japan).

Tensile strength was calculated from the tablet hardness (P_t_), punch diameter (r), and tablet thickness (h) as described in Equation (6):

Tensile strength (MPa) = 2P_t_/(πrh) (6)

All experiments were performed in quintuplicate.

### 3.10. Effect of the Amount of Added Water and Water Activity on the Disintegration Time and Tablet Hardness

Two-dimensional maps of the effects of the amount of added water and water activity on the disintegration time and tablet hardness, respectively, were created using OriginPRO (Version 2021b, OriginLab Corporation, Northampton, MA, USA.). A scatter plot was created to interpolate the data of disintegration time, hardness, and water activity from the amount of water added. Therefore, the connection line of the scatter plot was made into a B-spline, and the values were written in a table, which was used as the data after interpolation. In creating contour lines, the *X*-axis was fixed at the amount of water added, the *Y*-axis at the water activity, and the *Z*-axis at the hardness and decay time, and two types of contour lines were created. The contour lines were set as layer boundaries.

### 3.11. Statistical Analyses

Results are expressed as the mean ± standard deviation of three independent experiments. Two-group comparisons were performed using Student’s *t*-tests. Multiple-group comparisons were performed using one-way analysis of variance (ANOVA) and Fisher’s least-significant difference. Two-way ANOVA were performed on the data of manufacturing properties measured by Gamlen Development Series: D500. The data analysis was conducted using JMP^®^ pro 14 statistical software (SAS Institute, Cary, NC, USA). Differences in sample (X1) and loading weight (X2) were used as explanatory variables for this analysis, and *p*-values of <0.05 or 0.01 were considered statistically significant.

## 4. Conclusions

MADG has significant advantages over conventional methods in terms of reducing manufacturing energy and cost. In this study, we attempted to optimize manufacturing conditions of MADG to improve the tabletability of erythritol, which is an excipient for ODTs. MADG produced tablets with softer granules and larger voids than other granulation methods, indicating high hardness and rapid disintegration. This result confirms that MADG is a suitable granulation method for ODTs by improving the tabletability of erythritol. ODTs were prepared by varying the amount of water added in MADG and evaluated for disintegration time and hardness. When the amount of water added was increased, the disintegration time was prolonged because of the decrease in capillary wetting. If the water content was too high, the granules may agglomerate and the hardness may decrease. Finally, we optimized the manufacturing conditions for MADG, focusing on the amount of water added and water activity. It was determined that the water activity could be adjusted by selecting an additive that considers hygroscopicity. Tablets with desired hardness and disintegration properties could be produced by adjusting the amount of water to be added to approximately 2.7% and the water activity to approximately 0.4. These results are expected to be useful for the application of MADG as a new granulation method for ODTs.

## Figures and Tables

**Table 1:** Tablet formulation used in this study (% of tablet mass).

Process		MADG	HSG
Process Stage	Composition	-	-
Add Water (%) ^a^		1–5	15
1	Erythritol	70.0	70.0
	Povidone K25	-	4.0
	Povidone K12	4.0	-
	Avicel PH102	-	20.0
2	Avicel 200LM ^b^	20.0	-
3	Crospovidone XL	5.0	5.0
	Magnesium stearate	1.0	1.0
Total		100.0	100.0

1 = agglomeration/massing; 2 = moisture absorption; 3 = main and final blending; ^a^ liquid-to-powder ratio (volume of added water); ^b^ moisture absorbents.

## Data Availability

Data is contained within article and Supplementary Materials.

## Supplementary Materials

The following supporting information can be downloaded at https://www.mdpi.com/article/10.3390/ph15081004/s1, Figure S1: SEM photographs of the granules by MADG and HSG; Figure S2: SEM photographs of the inner structure of the tablets by MADG and HSG; Figure S3: Effect of the volume of water on the water activity of granules; Figure S4: Water activity of excipients. The bar indicates average ± S.D., *n* = 3; Figure S5: Photographs of the tables by MADG with amount of added water at 1% (left) and at 5% (right); Figure S6: Differences in tablet hardness between 2% and 4% added water. The bar indicates average ± S.D., *n* = 3; Table S1: Analysis of valiance (ANOVA) table for (**a**) compression pressure, (**b**) detachment stress, (**c**) ejection stress, (**d**) tensile strength, and (**e**) tablet density; Figure S7. Effect leverage plots for (**a**) compression pressure, (**b**) detachment stress, (**c**) ejection stress, (**d**) tensile strength, and (**e**) tablet density.

## References

[1] Lindgren S., Janzon L. (1991). Prevalence of swallowing complaints and clinical findings among 50–79-year-old men and women in an urban population. Dysphagia.

[2] Cook I.J., Kahrilas P.J. (1999). AGA technical review on management of oropharyngeal dysphagia. Gastroenterology.

[3] Barczi S.R., Sullivan P.A., Robbins J. (2000). How should dysphagia care of older adults differ? Establishing optimal practice patterns. Semin. Speech Lang..

[4] Schiele J.T., Quinzler R., Klimm H.D., Pruszydlo M.G., Haefeli W.E. (2013). Difficulties swallowing solid oral dosage forms in a general practice population: Prevalence, causes, and relationship to dosage forms. Eur. J. Clin. Pharmacol..

[5] Habib W., Khankari R., Hontz J. (2000). Fast-dissolve drug delivery systems. Crit. Rev. Ther. Drug Carr. Syst..

[6] Sugimoto M., Matsubara K., Koida Y., Kobayashi M. (2001). The preparation of rapidly disintegrating tablets in the mouth. Pharm. Dev. Technol..

[7] Almukainzi M., Araujo G.L.B., Löbenberg R. (2018). Orally disintegrating dosage forms. J. Pharm. Investig..

[8] FDA (2008). Guidance for Industry Orally Disintegrating Tablets.

[9] Badawy S.I., Menning M.M., Gorko M.A., Gilbert D.L. (2000). Effect of process parameters on compressibility of granulation manufactured in a high-shear mixer. Int. J. Pharm..

[10] Badawy S.I., Narang A.S., LaMarche K., Subramanian G., Varia S.A. (2012). Mechanistic basis for the effects of process parameters on quality attributes in high shear wet granulation. Int. J. Pharm..

[11] Pandey P., Tao J., Chaudhury A., Ramachandran R., Gao J.Z., Bindra D.S. (2013). A combined experimental and modeling approach to study the effects of high-shear wet granulation process parameters on granule characteristics. Pharm. Dev. Technol..

[12] Shi L., Feng Y., Sun C.C. (2010). Roles of granule size in over-granulation during high shear wet granulation. J. Pharm. Sci..

[13] Armstrong N.A. (1997). Selection of excipients for direct compression tablet formulation. Pharm. Technol. Eur..

[14] Rubinstein M.H. (1988). Tablets. Pharmaceutics: The Science of Dosage Form Design.

[15] Jivraj I.I., Martini L.G., Thomson C.M. (2000). An overview of the different excipients useful for the direct compression of tablets. Pharm. Sci. Technol. Today.

[16] Jarvinen M.A., Paaso J., Paavola M., Leiviska K., Juuti M., Muzzio F., Jarvinen K. (2013). Continuous direct tablet compression: Effects of impeller rotation rate, total feed rate and drug content on the tablet properties and drug release. Drug Dev. Ind. Pharm..

[17] Augsburger L.L., Zellhofer M.J. (2013). Tablet formulation. Encyclopedia of Pharmaceutical Science and Technology.

[18] Garg N., Pandey P., Kaushik D., Dureja H. (2015). Development of novel multifunction directly compressible co-processed excipient by melt granulation technique. Int. J. Pharm. Investig..

[19] Mangal S., Meiser F., Morton D., Larson I. (2015). Particle Engineering of Excipients for Direct Compression: Understanding the Role of Material Properties. Curr. Pharm. Des..

[20] Kunnath K., Huang Z., Chen L., Zheng K., Dave R. (2018). Improved properties of fine active pharmaceutical ingredient powder blends and tablets at high drug loading via dry particle coating. Int. J. Pharm..

[21] Otsuka A. (1998). Adhesive properties and related phenomena for powdered pharmaceuticals. Yakugaku Zasshi.

[22] Ullah I., Corrao R., Wiley G., Lipper R. (1987). Moisture activated dry granulation: A general process. Pharm. Technol..

[23] Ullah I., Chang J.W.Y., Wiley G.J., Jain N.B., Kiang S. (2009). Moisture-activated dry granulation part I: A guide to excipient and equipment selection and-formulation development. Pharm. Technol..

[24] Ullah I., Wang J., Chang S.Y., Guo H., Kiang S., Jain N.B. (2009). Moisture-activated dry granulation part II: The effects of formulation ingredients and manufacturing-process variables on granulation quality attributes. Pharm. Technol..

[25] Takasaki H., Yonemochi E., Messerschmid R., Ito M., Wada K., Terada K. (2013). Importance of excipient wettability on tablet characteristics prepared by moisture activated dry granulation (MADG). Int. J. Pharm..

[26] Thapa P., Lee A.R., Choi D.H., Jeong S.H. (2017). Effects of moisture content and compression pressure of various deforming granules on the physical properties of tablets. Powder Technol..

[27] Christensen L.H., Johansen H.E., Schaefer T. (2008). Moisture-Activated dry Granulation in a high Shear Mixer. Drug Dev. Ind. Pharm..

[28] Malamataris S., Goidas P., Dimitriou A. (1991). Moisture sorption and tensile strength of some tableted direct compression excipients. Int. J. Pharm..

[29] Railkar A.M., Schwartz J.B. (2000). Evaluation and comparison of a moist granulation technique to conventional methods. Drug Dev. Ind. Pharm..

[30] Railkar A.M., Schwartz J.B. (2001). The effects of formulation factors on the moist granulation technique for controlled-release tablets. Drug Dev. Ind. Pharm..

[31] Chen C.-M., Alli D., Igga M.R., Czeisler J.L. (2008). Comparison of Moisture-Activated Dry Granulation Profess with Conventional Granulation Methods for Sematilide Hydrochloride Tablets. Drug Dev. Ind. Pharm..

[32] Moravkar K.K., Ali T.M., Pawar J.N., Amin P.D. (2017). Application of moisture activated dry granulation (MADG) process to develop high dose immediate release (IR) formulations. Adv. Powder Technol..

[33] Takasaki H., Yonemochi E., Ito M., Wada K., Terada K. (2016). The effect of water activity on granule characteristics and tablet properties produced by moisture activated dry granulation (MADG). Powder Technol..

[34] Aodah A.H., Fayed M.H., Alalaiwe A., Alsulays B.B., Aldawsari M.F., Khafagy E.S. (2020). Design, Optimization, and Correlation of in vitro/in vivo Disintegration of Novel Fast Orally Disintegrating Tablet of High Dose Metformin Hydrochloride Using Moisture Activated Dry Granulation Process and Quality by Design Approach. Pharmaceutics.

[35] Kuno Y., Kojima M., Ando S., Nakagami H. (2008). Effect of preparation method on properties of orally disintegrating tablets made by phase transition. Int. J. Pharm..

[36] Tanimura S., Tahara K., Takeuchi H. (2015). Spray-dried composite particles of erythritol and porous silica for orally disintegrating tablets prepared by direct tableting. Powder Technol..

[37] Okuda Y., Irisawa Y., Okimoto K., Osawa T., Yamashita S. (2009). A new formulation for orally disintegrating tablets using a suspension spray-coating method. Int. J. Pharm..

[38] Mizumoto T., Masuda Y., Yamamoto T., Yonemochi E., Terada K. (2005). Formulation design of a novel fast-disintegrating tablet. Int. J. Pharm..

[39] Bi Y.X., Sunada H., Yonezawa Y., Danjo K. (1999). Evaluation of rapidly disintegrating tablets prepared by a direct compression method. Drug Dev. Ind. Pharm..

[40] Takasaki H., Sakurai A., Katayama T., Matsuura Y., Ohyagi N., Wada K., Ishikawa A., Yonemochi E. (2019). Novel, lean and environment-friendly granulation method: Green fluidized bed granulation (GFBG). Int. J. Pharm..

[41] Fukami J., Yonemochi E., Yoshihashi Y., Terada K. (2006). Evaluation of rapidly disintegrating tablets containing glycine and carboxymethylcellulose. Int. J. Pharm..

[42] Badawy S.I., Gray D.B., Hussain M.A. (2006). A study on the effect of wet granulation on microcrystalline cellulose particle structure and performance. Pharm. Res..

[43] Snider B., Liang P., Pearson N. (2007). Implementation of water-activity testing to replace Karl Fischer water testing. Pharm. Technol..

[44] Abdel-Hamid S., Betz G. (2011). Radial die-wall pressure as a reliable tool for studying the effect of powder water activity on high speed tableting. Int. J. Pharm..

[45] Roopwani R., Buckner I.S. (2019). Co-Processed Particles: An Approach to Transform Poor Tableting Properties. J. Pharm. Sci..

[46] Zhang Y., Law Y., Chakrabarti S. (2003). Physical properties and compact analysis of commonly used direct compression binders. AAPS PharmSciTech.

[47] Osamura T., Takeuchi Y., Onodera R., Kitamura M., Takahashi Y., Tahara K., Takeuchi H. (2016). Characterization of tableting properties measured with a multi-functional compaction instrument for several pharmaceutical excipients and actual tablet formulations. Int. J. Pharm..

[48] Medarevic D., Djuris J., Krkobabic M., Ibric S. (2021). Improving Tableting Performance of Lactose Monohydrate by Fluid-Bed Melt Granulation Co-Processing. Pharmaceutics.

[49] Dular Vovko A., Hodžić B., Brec T., Hudovornik G., Vrečer F. (2022). Influence of Formulation Factors, Process Parameters, and Selected Quality Attributes on Carvedilol Release from Roller-Compacted Hypromellose-Based Matrix Tablets. Pharmaceutics.

[50] Fell J.T., Newton J.M. (1970). Determination of tablet strength by the diametral-compression test. J. Pharm. Sci..

