# Supplementary material for: Study of Orally Disintegrating Tablets Using Erythritol as an Excipient Produced by Moisture-Activated Dry Granulation (MADG)
Source: Pharmaceuticals (Basel). 2022 Aug 15;15(8):1004. doi: 10.3390/ph15081004 (PMC9415806; doi:10.3390/ph15081004)
Supplement: Supplementary file 1 [file pharmaceuticals-15-01004-s001.zip › pharmaceuticals-1863531-supplementary.pdf]

pharmaceuticals

Supporting Materials

Study of orally disintegrating tablets using erythritol as an excipient produced by moisture-activated dry granulation (MADG)

Mizuki Yamada[1,†], Agata Ishikawa [1,†], Shun Muramatsu [1], Takayuki Furuishi[1,*], Yoshinori Onuki[2], Kaori Fukuzawa[1,3], Etsuo Yonemochi[1,*]

[1] Department of Physical Chemistry, School of Pharmacy and Pharmaceutical Sciences, Hoshi University, 2-4-41 Ebara, Shinagawa-ku, 142-8501 Tokyo, Japan

[2] Laboratory of Pharmaceutical Technology, School of Pharmacy and Pharmaceutical Sciences, University of Toyama, 2630 Sugitani, Toyama-shi, Toyama, 930-0194, Japan

[3] Graduate School of Pharmaceutical Sciences, Osaka University; 1-6 Yamadaoka, Suita, 565-0871, Osaka Japan

* Correspondence: t-furuishi@hoshi.ac.jp (T.F.), e-yonemochi@hoshi.ac.jp (E.Y.)

† These authors contributed equally to this work.

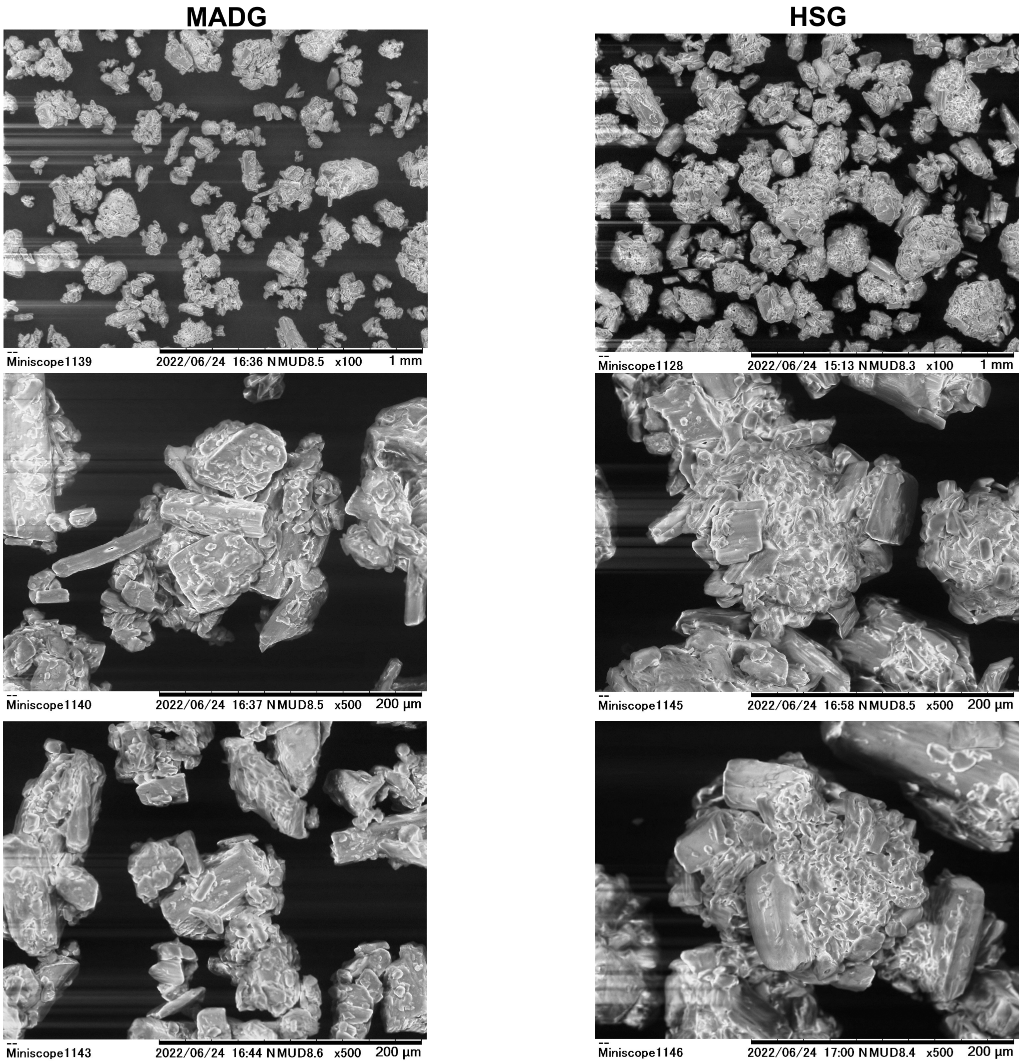

Figure S1. SEM photographs of the granules by MADG and HSG

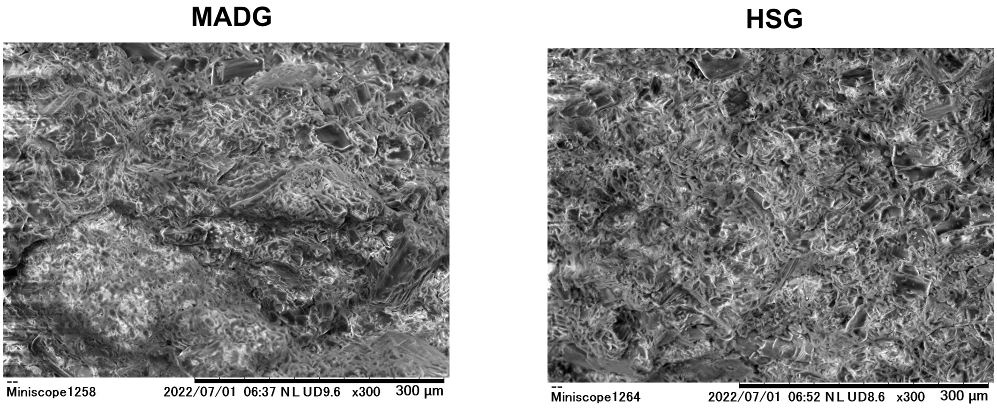

Figure S2. SEM photographs of the inner structure of the tablets by MADG and HSG

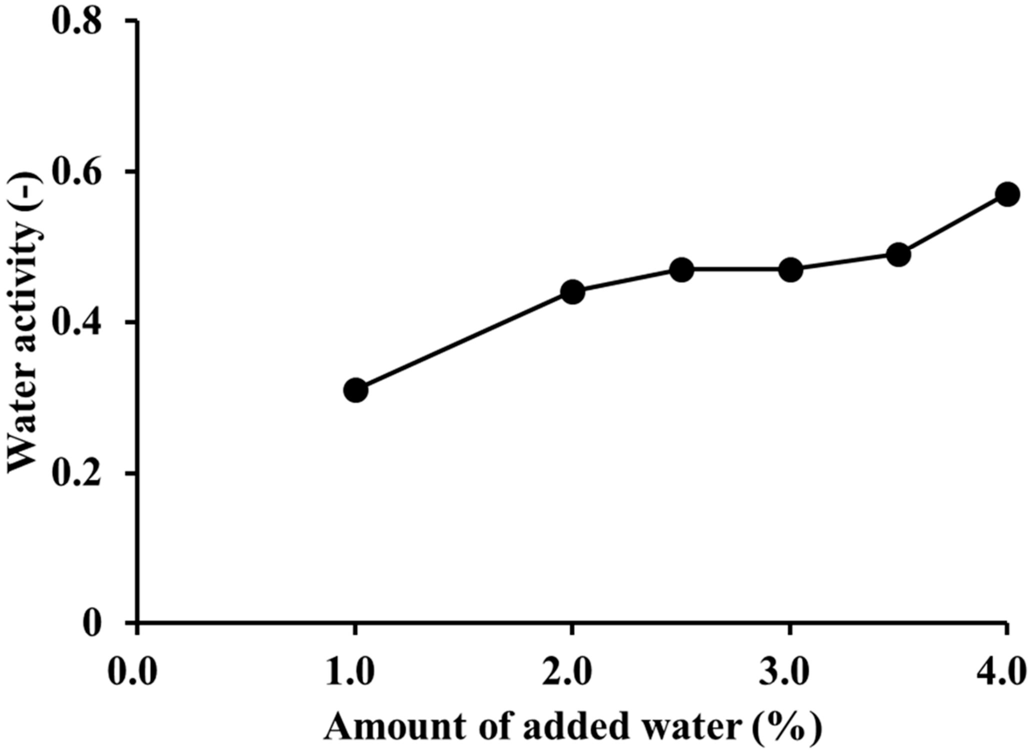

Figure S3. Effect of the volume of water on the water activity of granules

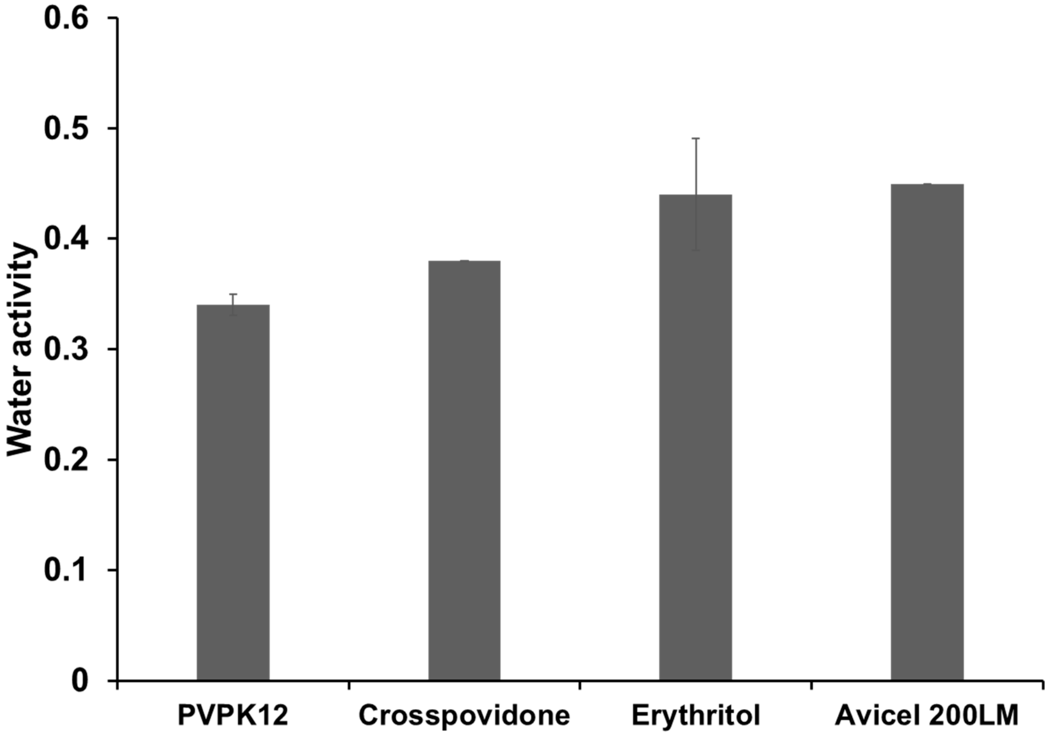

Figure S4. Water activity of excipients. The bar indicates average ± S.D., $n = 3$

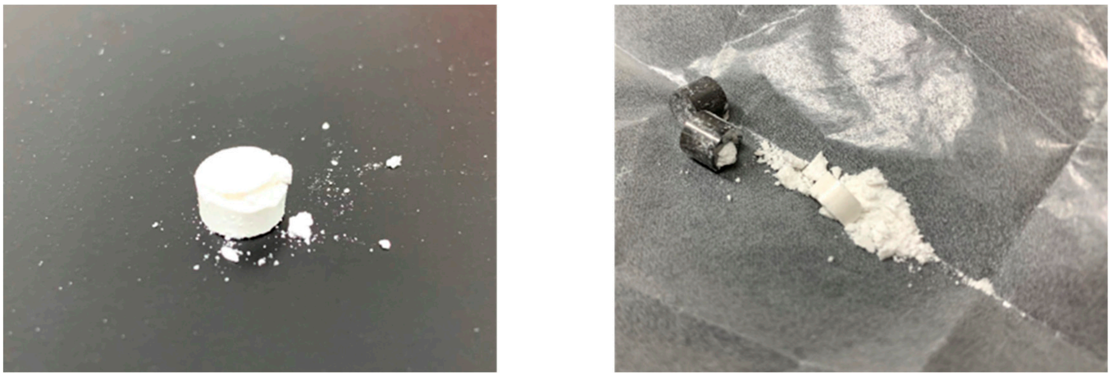

Figure S5. Photographs of the tables produced using MADG with 1% added water (left) and at 5% added water (right).

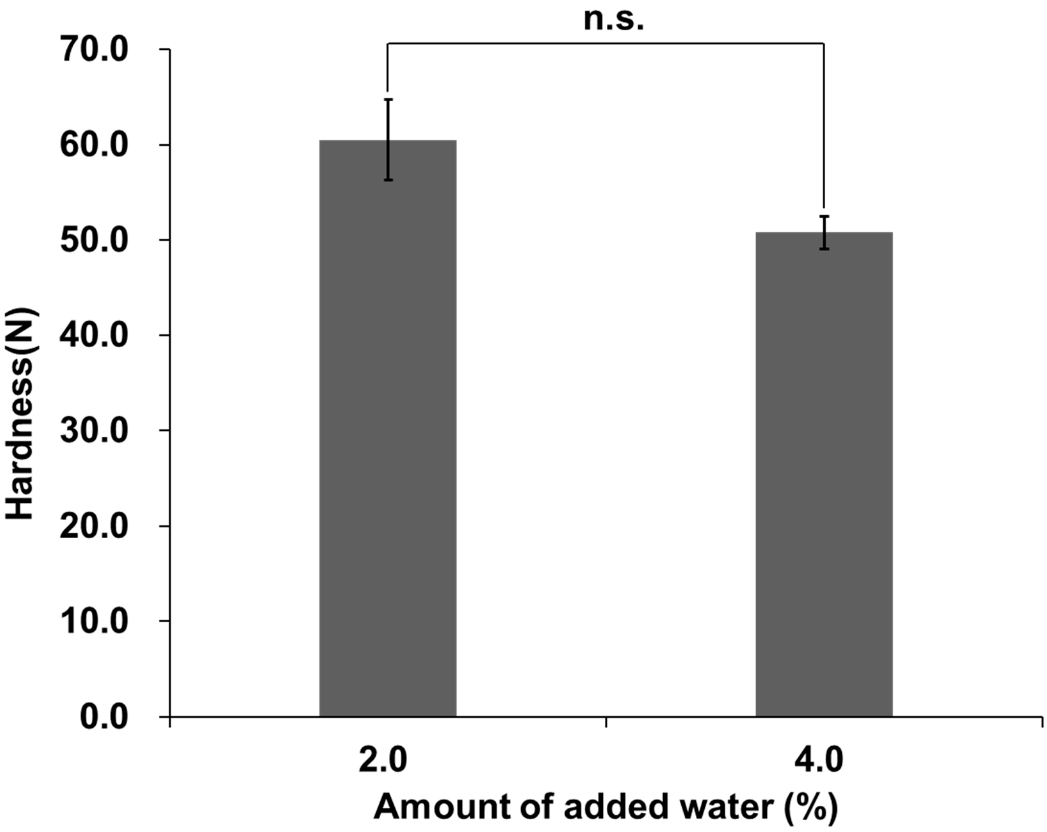

Figure S6. Differences in tablet hardness between 2% and 4% added water The bar indicates average ± S.D., n = 3

Table S1. Analysis of valiance (ANOVA) table for (a) compression pressure, (b) detachment stress, (c) ejection stress, (d) tensile strength, and (e) tablet density

(a)

	DF[a]	MS[b]	*F*o[c]	*P* value
X1: Sample	1	0.1	4.0	0.0561
X2: Loading weight	2	59684.2	1002161.5	< 0.01
Error	26	0.8		
Total	29	59685.1		

[a] Degree of freedom, [b] Mean square, [c] Observed *F* value

(b)

	DF[a]	MS[b]	*F*o[c]	*P* value
X1: Sample	1	7.4	121.7	< 0.01
X2: Loading weight	2	59.2	484.5	< 0.01
Error	26	1.6		
Total	29	68.3		

[a] Degree of freedom, [b] Mean square, [c] Observed *F* value

(c)

	DF[a]	MS[b]	*F*o[c]	*P* value
X1: Sample	1	4.6	53.1	< 0.01
X2: Loading weight	2	13.7	78.8	< 0.01
Error	26	2.3		
Total	29	20.6		

[a] Degree of freedom, [b] Mean square, [c] Observed *F* value

(d)

	DF[a]	MS[b]	*F*o[c]	*P* value
X1: Sample	1	0.65	26.9	< 0.01
X2: Loading weight	2	12.16	251.7	< 0.01
Error	26	0.63		
Total	29	13.44		

[a] Degree of freedom, [b] Mean square, [c] Observed *F* value

(e)

	DF[a]	MS[b]	*F*o[c]	*P* value
X1: Sample	1	0.004	7.0	< 0.05
X2: Loading weight	2	0.077	63.5	< 0.01
Error	26	0.016		
Total	29	0.097		

[a] Degree of freedom, [b] Mean square, [c] Observed *F* value

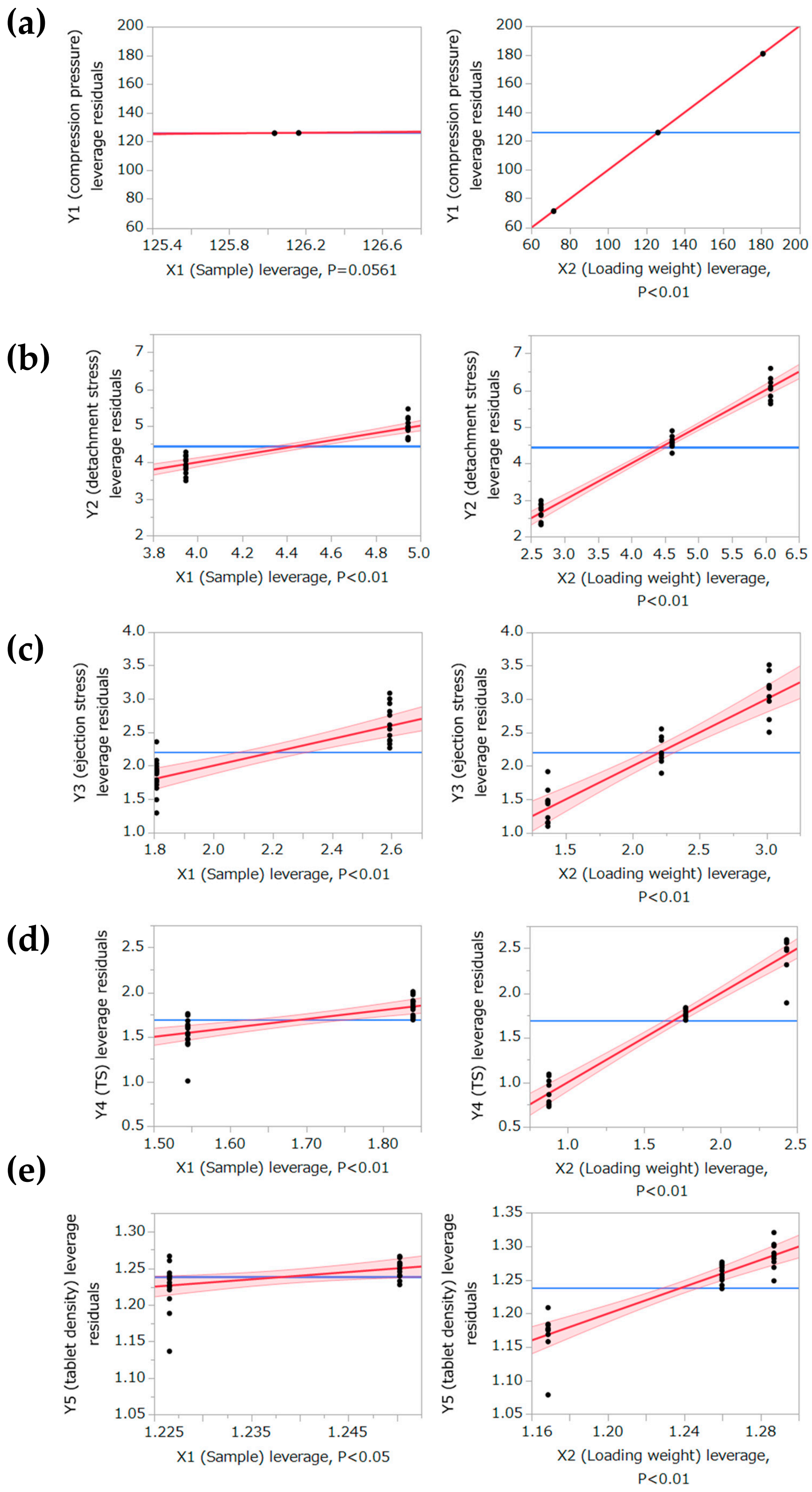

Figure S7. Effect leverage plots for (a) compression pressure, (b) detachment stress, (c) ejection stress, (d) tensile strength, and (e) tablet density
